# Geometrical Bounds on Irreversibility in Squeezed Thermal Bath

**DOI:** 10.3390/e25010128

**Published:** 2023-01-09

**Authors:** Chen-Juan Zou, Yue Li, Jia-Kun Xu, Jia-Bin You, Ching Eng Png, Wan-Li Yang

**Affiliations:** 1 Research Center of Nonlinear Science, School of Mathematical and Physical Science, Wuhan Textile University, Wuhan 430200, China; cjzou@hunnu.edu.cn; 2 State Key Laboratory of Magnetic Resonance and Atomic and Molecular Physics, Innovation Academy for Precision Measurement Science and Technology, Chinese Academy of Sciences, Wuhan 430071, China; liyue6683@gmail.com (Y.L.); ywl@wipm.ac.cn (W.-L.Y.); 3 School of Physical Sciences, University of Chinese Academy of Sciences, Beijing 100049, China; 4 Institute of High Performance Computing, Agency for Science, Technology, and Research (A*STAR), 1 Fusionopolis Way, #16-16 Connexis, Singapore 138632, Singapore; pngce@ihpc.a-star.edu.sg

**Keywords:** squeezed thermal bath, irreversible entropy production, geometrical bounds

## Abstract

Irreversible entropy production (IEP) plays an important role in quantum thermodynamic processes. Here, we investigate the geometrical bounds of IEP in nonequilibrium thermodynamics by exemplifying a system coupled to a squeezed thermal bath subject to dissipation and dephasing, respectively. We find that the geometrical bounds of the IEP always shift in a contrary way under dissipation and dephasing, where the lower and upper bounds turning to be tighter occur in the situation of dephasing and dissipation, respectively. However, either under dissipation or under dephasing, we may reduce both the critical time of the IEP itself and the critical time of the bounds for reaching an equilibrium by harvesting the benefits of squeezing effects in which the values of the IEP, quantifying the degree of thermodynamic irreversibility, also become smaller. Therefore, due to the nonequilibrium nature of the squeezed thermal bath, the system–bath interaction energy has a prominent impact on the IEP, leading to tightness of its bounds. Our results are not contradictory with the second law of thermodynamics by involving squeezing of the bath as an available resource, which can improve the performance of quantum thermodynamic devices.

## 1. Introduction

Over the past two decades, the nonequilibrium phenomena and thermodynamic irreversibility quantified by irreversible entropy production (IEP) have drawn much attention, since this fundamental concept is one of the cornerstones of classical and quantum thermodynamics [1,2,3,4,5,6,7,8,9,10]. As is well-known, the positivity of entropy production has been universally captured by the conventional second law of thermodynamics (SLT) [9], which quantitatively characterizes the interplay between the exchange of energy and the irreversibility by introducing the state function entropy [11,12]. In addition, through specifying a lower bound for the irreversible entropy change, the related Clausius inequality provides a fundamental feature of irreversible phenomena. Note that this lower bound (zero) is trivially independent of how far from equilibrium a process operates [6].

Recently, with restriction to a specific class of nonequilibrium phenomena, such as thermal relaxation process, rich features of thermodynamic irreversibility have been found successively [2]. Such a lower bound for classical, near-equilibrium transformation processes has been derived by means of a geometric approach [13]. S. Deffner and E. Lutz treated the system’s Hilbert space as a Riemannian manifold and extended the classical case [13] to the nonequilibrium closed quantum system. The obtained generalized Clausius inequality states that the thermodynamic irreversibility is bounded in terms of the Bures length between the final state and the corresponding equilibrium state by using information geometry [6].Soon afterwards, they broadened the closed quantum system further to the weakly coupled open quantum system, then obtained the exact microscopic expressions for the nonequilibrium entropy production [7]. Along this direction, reference [11] theoretically and experimentally determined a sharper geometrical bound for a qubit thermalization process and obtained a tighter version of the Clausius inequality following a similar approach.

In many cases of interest, however, having a sharper lower bound is essential. A case in point is the optimization of the performance of real finite-time thermodynamic processes [14,15]. Therefore, the abovementioned progress stimulated successive studies on the tightness of the geometrical bounds on irreversibility in open quantum systems [6,7,8,9,10,11]. These recent publications tried to develop theories to further understand the thermodynamic irreversibility inherent to nonequilibrium processes. In particular, based on the variational principle and time-reversed map, the authors in reference [10] obtained an information–theoretical bound for entropy production in a relaxation process by a geometric distance on the Riemannian manifold [16], which was experimentally validated by a single ultracold trapped ion ^40^Ca [17].

T. V. Vu and Y. Hasegawa strengthened the Clausius inequality and proved that IEP is bounded from below by a modified Wasserstein distance (quantum generalization of the Wasserstein metric) between the initial and final states [8]. Thereafter, they extended this single-bath case to the case of multiple-bath, and refined the bound in a quantum regime, through deriving the fundamental bound on irreversibility for thermal relaxation processes of Markovian open quantum systems [9]. 

**Figure 1:**
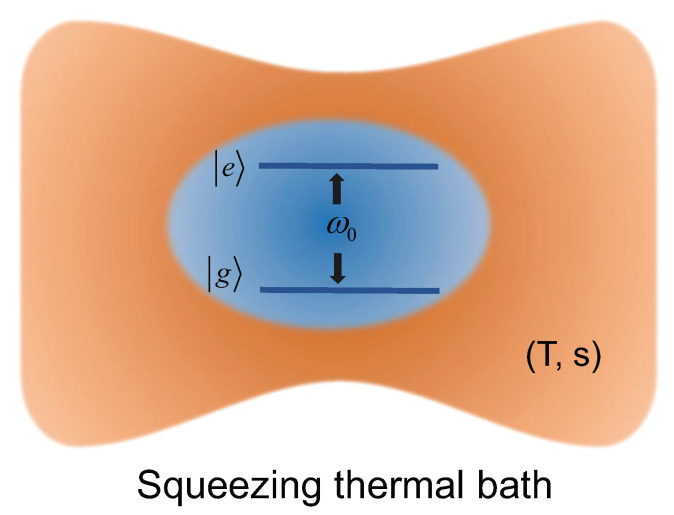
Schematic diagram for two-level system (excited state $|e\rangle$ and ground state $|g\rangle$ ) interacting with a squeezed thermal bath at temperature *T* with squeezing parameter *s*. The transition frequency between the two levels is $\omega_{0}$ . The evolution induced by the system-bath interaction produces irreversible entropy.

On the other hand, quantum bath engineering techniques are powerful tools that enable the realization of arbitrary thermal and nonthermal environments [18,19,20,21,22,23,24,25]. Additionally, due to the unique vantage of the quantum control, bath engineering has aroused widespread interest in the context of quantum thermodynamical processes. Various strategies have attempted to improve the performance of thermodynamic devices [26,27,28,29,30,31,32,33,34], whose efficiency is usually reduced by the presence of IEP [7]. For instance, the use of a squeezed thermal bath as shown in Figure 1 allows us to operate thermodynamic devices beyond the classical bound [25,26]. In particular, the experiment in reference [32] demonstrated that the efficiency of the quantum heat engine may go beyond the standard Carnot efficiency by employing a squeezed thermal bath.

However, the influence of squeezed thermal bath on the bounds of IEP in the quantum thermodynamics has been largely unexplored. A precise characterization of the IEP in such an uncharted domain, and a general framework providing a deeper understanding of the associated quantum thermodynamic phenomena therefore appear necessary. Therefore, it is instructive to look into the role th squeezed thermal bath plays during the process of thermodynamic irreversibility.

In this work, we study and quantify the geometrical bounds on irreversibility of a quantum system in contact with a squeezed thermal bath. Here, we consider two distinct situations, including the dissipation model and dephasing model, respectively. Starting with the Born–Markovian quantum master equation in the weak coupling limit, we derived analytical expressions for time-dependent reduced density matrix of system, in which the squeezing parameter of the bath is involved. Then, through quantifying the degree of irreversibility by the IEP, we find that the geometrical bounds of IEP decrease (increase) with the growth of degree of squeezing in the case of the dissipation (dephasing) model, respectively. Additionally, the common feature is that the critical times of the IEP itself and that of IEP’s bounds reaching equilibrium, as well as the values of IEP quantifying the degree of thermodynamic irreversibility are reduced due to the presence of the squeezing effect for these two models. Therefore, due to the nonequilibrium nature of a squeezed thermal bath, the interaction energy between the system and the bath brought an important impact on the irreversibility, as well as the tightness of its bounds. As expected, our finding obeys the principles of thermodynamics and reveals richer features of the thermodynamics relaxation process, and the presence of a quantum property, such as the squeezing effects included in the bath, could be used to serve as an available resource to improve the performance of the quantum thermodynamic devices.

The paper is organized as follows. We give a brief account of the method about geometrical bounds on irreversibility in an open quantum system (Section 2). We evaluate the geometrical bounds on irreversibility for a squeezed thermal bath in the dissipation model (Section 3.1) and dephasing model (Section 3.2), respectively, and we conclude and give prospects for future developments in Section 4.

## 2. Materials and Methods

### Geometrical Bounds of Irreversible Entropy Production

Considering an arbitrary quantum system with the Hamiltonian coupled to a thermal bath, the quantum system is usually initialized in a given state $\rho_{0}$ , then interacts with a bath at temperature *T*. The evolution induced by the interactions brings the system in a state ρ(t) and produces irreversible entropy. Furthermore, the system will thermalize with the bath and then asymptotically reach the unique canonical equilibrium state $\rho_{th}$ if the system Hamiltonian *H* remains constant. The total entropy variation of the system is defined as

(1) $$\Delta S_{tot}=\Delta S_{ir}+\Delta S_{re}=S\left[\rho (t)\right]-S\left[\rho_{0}\right],$$

where $S\left[\rho \right]=-tr\left(\rho \ln\rho \right)$ is the von Neumann entropy. The IEP (irreversible part of the total entropy variation $\Delta S_{tot}$ ) is denoted by

(2) $$\Delta S_{ir}\left(t\right)=S\left(\rho_{0}∥\rho_{th}\right)-S\left(\rho \left(t\right)∥\rho_{th}\right),$$

which is the thermodynamic irreversibility under consideration in the present work [2,11]. $S\left(\rho_{1}∥\rho_{2}\right)=tr\left(\rho_{1}\ln\rho_{1}\right)-tr\left(\rho_{1}\ln\rho_{2}\right)$ represents the quantum relative entropy of $\rho_{1}$ to $\rho_{2}$ . The entropy flow between the system and the environment (reversible part of the total entropy variation $\Delta S_{tot}$ ) is $\Delta S_{re}=\Delta Q/T$ , where $\Delta Q=tr\left(H\rho (t)\right)-tr\left(H\rho_{0}\right)$ is the heat absorbed by the system [11]. On the other hand, the Clausius inequality $\Delta S_{ir}\geq 0$ putting forward the lower limit of IEP is always non-negative. In order to deepen the understanding of how much energy in the irreversible process is consumed, it is essential to search a sharper or tighter bound on irreversibility. By treating the Hilbert space of the system as a Riemannian manifold, the relationship between IEP and the geodesic distance *D* corresponding to the metric that is contractive under complete positive and trace preserving maps can be directly established, and a generalized form of the Clausius inequality can be obtained by deriving the Wigner–Yanase length between the initial and final states of the system. Note that the only cases in which an analytical expression for the geodesic distance is known are the Wigner-Yanase metric $D_{WY}\left(\rho_{1},\rho_{2}\right)=\arccos\left[tr\left\{\sqrt{\rho_{1}}\sqrt{\rho_{2}}\right\}\right]$ and the quantum Fisher information metric $D_{QF}\left(\rho_{1},\rho_{2}\right)=\arccos\left[tr\left\{\sqrt{\sqrt{\rho_{1}}\rho_{2}\sqrt{\rho_{1}}}\right\}\right]$ [11]. Based on these analytical expressions, one can obtain the geometric lower bound (LB) of IEP as [11,35,36]

(3) $$\Delta S_{ir}\left(t\right)\geq \frac{8}{\pi^{2}}\max_{\left\{X=QF,WY\right\}}D_{X}^{2}\left(\rho_{0},\rho \left(t\right)\right),$$

and the geometrical upper bound (UB) is

(4) $$\Delta S_{ir}\left(t\right)\leq S\left(\rho_{0}∥\rho_{th}\right)-\frac{8}{\pi^{2}}\max_{\left\{X=QF,WY\right\}}D_{X}^{2}\left(\rho \left(t\right),\rho_{th}\right).$$

From the above two relations, one can define the related bound gap as

(5) ΔU=UB−LB,

and the deviation of the IEP from the LB or UB is given by

(6) $$\Delta \delta_{L}=\Delta S_{ir}\left(t\right)-LB,$$

or

(7) $$\Delta \delta_{U}=UB-\Delta S_{ir}\left(t\right),$$

respectively. In this paper, we say an LB (UB) is relatively tighter if the LB (UB) takes a larger (smaller) value compared with the case of a conventional thermal bath.

## 3. Results

### 3.1. Geometrical Bounds on Irreversibility in the Dissipation Model

Here we consider the dissipation model, taking into account the effect of the squeezed thermal bath with temperature *T* in the case of single-excitation. The total Hamiltonian is (in units of ℏ=1 )

(8) $$\begin{array}{ccc} H & = & H_{S}+H_{B}+H_{SB}\\ & = & \omega_{0}{\hat{\sigma }}_{+}{\hat{\sigma }}_{-}+\sum_{k}\omega_{k}{\hat{b}}_{k}^{†}{\hat{b}}_{k}+\sum_{k}\left(g_{k}{\hat{\sigma }}_{+}{\hat{b}}_{k}+H.c.\right), \end{array}$$

where $H_{S}$ , $H_{B}$ , and $H_{SB}$ stand for the Hamiltonians of the system, bath, and system–bath interaction, respectively; ${\hat{\sigma }}_{+}\left({\hat{\sigma }}_{-}\right)=|e\rangle \langle g|\left(|g\rangle \langle e|\right)$ and $\omega_{0}$ are the inversion operator and transition frequency of the system with $|e\rangle$ and $|g\rangle$ being the excited and ground states; ${\hat{b}}_{k}^{†}\left({\hat{b}}_{k}\right)$ are the creation (annihilation) operators of the k-th mode of the bath. The coupling strength between the system and the bath is denoted by $g_{k}$ .

The master equation in the interaction picture is given by the following Lindblad form [37,38]

(9) $$\begin{array}{ccc} {\dot{\rho }}_{s}\left(t\right) & = & \frac{\gamma N}{2}D\left[\sigma_{+}\right]\rho_{s}\left(t\right)+\frac{\gamma \left(1+N\right)}{2}\left[\sigma_{-}\right]\rho_{s}\left(t\right)\\ & & -\gamma M\sigma_{+}\rho_{s}\left(t\right)\sigma_{+}-\gamma M^{*}\sigma_{-}\rho_{s}\left(t\right)\sigma_{-}, \end{array}$$

where ${\dot{\rho }}_{s}\left(t\right)=d\rho_{s}\left(t\right)/dt$ , and $D\left[A\right]\rho =2A\rho A^{+}-A^{+}A\rho -\rho A^{+}A$ . The spontaneous emission rate of the system is γ and $N=N_{th}\left[\cosh^{2}\left(s\right)+\sinh^{2}\left(s\right)\right]+\sinh^{2}\left(s\right)$ , and $M=-\sinh\left(2s\right)e^{i\phi }\left(2N_{th}+1\right)/2$ with *s* and ϕ the bath squeezing strength and phase, respectively. $N_{th}=1/\left(e^{\omega_{0}\beta }-1\right)$ is the Plank distribution, where $\beta =1/k_{B}T$ with $k_{B}=1$ the Boltzmann constant.

Rewriting the density matrix as $\rho_{s}\left(t\right)$ $=\left(I+\vec{r}\left(t\right)\cdot \vec{\sigma }\right)/2$ by means of the Bloch vector $\vec{r}\left(t\right)=Tr\left[\vec{\sigma }\rho_{s}\left(t\right)\right]$ with the identity matrix I , we can transform the master Equation (9) into the Bloch equation

(10) $$\frac{d}{dt}\vec{r}\left(t\right)=\xi \vec{r}\left(t\right)+\vec{m},$$

with

(11) $$\xi =\left(\begin{array}{ccc} -\frac{\tilde{\gamma }+2\gamma M}{2} & 0 & 0\\ 0 & -\frac{\tilde{\gamma }-2\gamma M}{2} & 0\\ 0 & 0 & -\tilde{\gamma } \end{array}\right)$$

and $\vec{m}={\left(0,0,-\gamma_{0}\right)}^{T}$ . Here, $\tilde{\gamma }=\gamma \left(2N+1\right)$ is the total transition rate. Assuming the system to be initially in the ground state $\left(|e\rangle +|g\rangle \right)/\sqrt{2}$ , a straightforward calculation yields the analytical solution

(12) $$\rho_{s}\left(t\right)=\left(\begin{array}{cc} \frac{1-\nu +\left(1-\nu \right){\langle \sigma_{z}\rangle }_{ss}}{2} & \mu {\langle \sigma_{-}\rangle }_{ss}\\ \left(1-e^{\frac{-\gamma_{s}t}{2}}\right){\langle \sigma_{+}\rangle }_{ss} & \frac{1+\nu -\left(1-\nu \right){\langle \sigma_{z}\rangle }_{ss}}{2} \end{array}\right),$$

Here, ${\langle \sigma_{\pm }\rangle }_{ss}$ and ${\langle \sigma_{z}\rangle }_{ss}\;$ are the stationary solutions of differential Equation (10); $\mu =\left\{\gamma_{s}-e^{\left(-4\tilde{\gamma }+\gamma_{s}\right)t/4}\left[\gamma_{s}\cos\left(\gamma_{s}t/4\right)+\left(\gamma_{s}+\gamma M\right)\sin\left(\gamma_{s}t/4\right)\right]\right\}/\gamma_{s}$ and $\nu =\tilde{\gamma }e^{\left(-4\tilde{\gamma }+\gamma_{s}\right)t/4}\left[\cos\left(\gamma_{s}t/4\right)-\sin\left(\gamma_{s}t/4\right)\right]/\gamma_{s}$ , where $\gamma_{s}=\tilde{\gamma }+2\gamma M$ .

**Figure 2:**
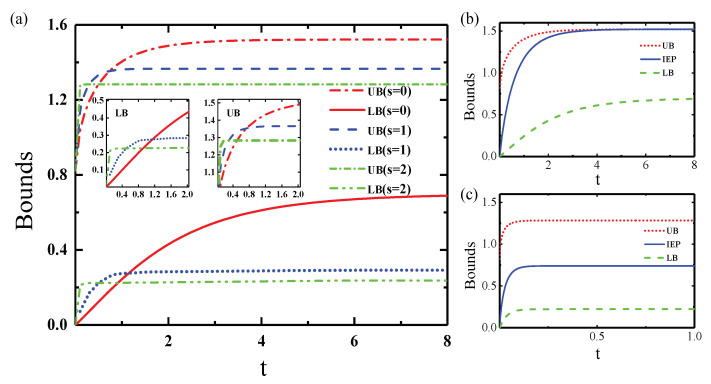
(**a**) Time dependence of LB and UB of IEP under different degree of squeezing *s* in the dissipation model. Time dependence of IEP (blue solid line) and its LB (red dotted line) and UB (green dashed line) in the case of (**b**) s=0 and (**c**) s=2 . The initial state of system is $\left(|e\rangle +|g\rangle \right)/\sqrt{2}$ . Hereafter, we choose $\omega_{0}$ as the frequency unit, T=0.34 , and ϕ=0.

The time dependence of the IEP and its geometrical bounds are explored by numerically calculating the quantities (Equations (2)–(4)) and plotted in Figure 2. It is clear from Figure 2 that IEP and its bounds (UB and LB) increase monotonically with time toward the corresponding equilibrium values for a squeezed thermal bath. For the non-squeezing case ( s=0 ), our results based on the quantum master equation are fully consistent with that for the thermal bath in reference [11] using the method of Kraus operators. In the case of a squeezed thermal bath ( s>0 ), with an increase in the degree of squeezing, the UB becomes higher in the early stage of evolution t≲0.15 and then becomes reduced with the growth of *s* when t≳0.45 , compared with the thermal bath ( s=0 ), as shown in Figure 2a. In addition, the dynamical behavior of LB shares similar features with that of UB. Another common feature between UB and LB is that their equilibrium times decrease with the growing degree of squeezing. For instance, the equilibrium times for UB (LB) are 4.5 (7.5), 2 (1.4), and 0.15 (0.2) when s=0 , 1, and 2, respectively. The physical picture is that the evolution time from the pure state to the maximum mixed state becomes shorter with the growth of squeezing parameter. We conclude that the summarized overall trends of geometrical bounds (LB and UB) found in reference [11] also hold for a squeezed thermal bath, while only the UB becomes tighter in the longtime limit, and the LB exhibits subtle tightness in the early stage of evolution compared with the traditional thermal bath [11].

Let us next examine the time dependence of the IEP. The numerical simulation plotted in Figure 2b,c suggests that the values of IEP are well bounded in the region between the LB and UB. Furthermore, the squeezed thermal bath has prominent influences in both the concrete values of IEP and the critical times $T_{c}$ of IEP reaching equilibrium, where the values of $T_{c}$ become less for growing values of *s*. Another observation in Figure 2b is that the deviation $\Delta \delta_{L}$ (Equation (6)) increases monotonically and gradually coincides with ΔU (Equation (5)), whereas the deviation $\Delta \delta_{U}$ (Equation (7)) decreases monotonically and gradually disappears to zero in the case of s=0 . With the increase of squeezing strength, i.e., s=2 , as shown in Figure 2c, the deviation $\Delta \delta_{L}$ ( $\Delta \delta_{U}$ ) increases (decreases) gradually to a fixed value 0.51 (0.542), and the rates of change of deviations $\Delta \delta_{L}$ and $\Delta \delta_{U}$ become slower. From the above results we deduce that the actual amount of IEP departs from its UB gradually and approaches its LB with the growth of *s*.

**Figure 3:**
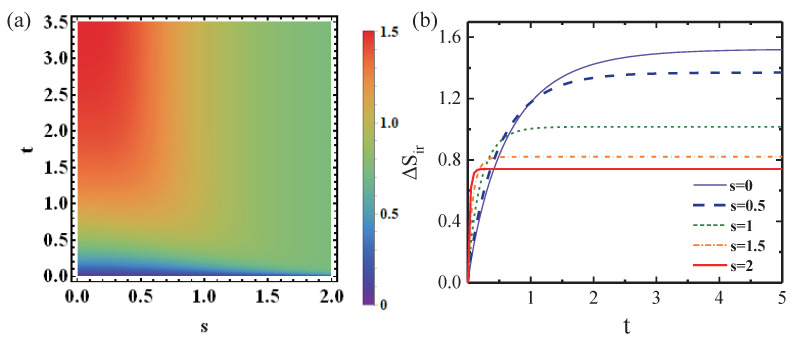
(**a**) The values of the IEP $▵S_{ir}$ in the parameter plane of {s,t} in the dissipation model; (**b**) time dependence of IEP $▵S_{ir}$ under the different degrees of squeezing *s*.

To obtain a clear picture of how the IEP evolves in the parameter space of {s,t} , we plot the evolution of IEP in Figure 3. It shows that the values of IEP are obviously dependent on the squeezing parameters of the bath. Here, we provide remarks on the parameter dependence. Although the values of *s* are not directly related to the system–bath interaction, they depend on both correlation time and occupation number of the bath and then immensely affect the relaxation dynamics and steady state of the relevant system during the thermodynamic process. As a result, any change of this key parameter will have significant influence on the irreversibility, and the inherent squeezing effect stemming from the bath plays a crucial role in understanding the relative tightness of the bounds. As we have shown analytically in the previous paragraphs, the above summarized dependence of bounds and IEP on the squeezing parameter is reflected in Equations (3), (4) and (12).

Traditionally, the IEP could be used to evaluate the performance of thermodynamic devices, such as the ergotropy (or energy) that can be extracted from a given system, and the maximal useful work, which is usually reduced by the presence of irreversibility [7,39]. It implies that the irreversibility can be restrained by controlling the amount of IEP through adjusting the degree of the squeezing effect, as shown in Figure 3b, where the values of IEP become less for growing values of *s*. From the perspective of quantum bath engineering, employing a squeezed thermal bath is a promising avenue of using a squeezing effect as a quantum resource to improve the performance of thermodynamic devices [25,26,32].

As we know, a squeezing effect that is rooted in Heisenberg’s uncertainty principle can be defined as the reduction in the uncertainty of some observable at the cost of the build-up in the conjugate one [28,40,41]. Physically, the squeezing involved in the bath thereby inevitably modifies the nonunitary relaxation dynamics of the system and the relevant irreversibility during the thermodynamic process. Compared with the thermal bath, the squeezed thermal bath is taken out of thermodynamic equilibrium through the squeezing operation, with the consequence that its excitation number changes from $N_{th}$ to $N=N_{th}\left(\cosh^{2}r+\sinh^{2}r\right)+\sinh^{2}r$ [28,42], which can be seen as an increase in its effective temperature $T_{eff}=\omega_{h}/k_{B}\ln\left[1/\left(N_{th}^{-1}+1\right)\right]$ with a higher frequency $\omega_{h}>\omega_{0}$ . Therefore, being purely quantum mechanical fuel in nature, a squeezed thermal bath is beneficial in its own way by providing us with more compact energy storage and a higher effective high temperature bath without actually being too hot [5]. That is to say, the squeezed thermal state has the same entropy as the Gibbs state, but increased mean energy, which is instrumental in the suppression of irreversibility.

### 3.2. Geometrical Bounds on Irreversibility in the Dephasing Model

Next, we focus on the dephasing model with respect to the squeezed thermal bath, where the bath operator is simply a sum of linear couplings to the coordinates of a continuum of harmonic oscillators described by a spectral density function J(ω) [43,44,45,46,47,48], and the decay of the coherence occurs without a decay of the corresponding populations. Now, the total Hamiltonian is

(13) $$\begin{array}{ccc} H & = & H_{S}+H_{B}+H_{SB}\\ & = & \omega_{0}{\hat{\sigma }}_{+}{\hat{\sigma }}_{-}+\sum_{k}\omega_{k}{\hat{b}}_{k}^{†}{\hat{b}}_{k}+{\hat{\sigma }}_{z}\sum_{k}\left(g_{k}{\hat{b}}_{k}+H.c.\right). \end{array}$$

The dynamics of the system can be characterized by the reduced density matrix which is obtained by tracing out the degrees of freedom of the bath. In the interaction picture, using the rotating wave approximation, the reduced density matrix of the system can be written as [46,47,48,49,50]

(14) $$\rho_{s}\left(t\right)=\left[\begin{array}{cc} \rho_{ee} & \rho_{eg}\Gamma \left(t\right)\\ \rho_{ge}\Gamma^{*}\left(t\right) & \rho_{gg} \end{array}\right],$$

where the phase decay behavior of the qubit under the influence of the bath is denoted by the factor $\Gamma \left(t\right)=Tr_{B}\rho_{B}\prod_{k}\exp\left[\alpha_{k}\left(t\right){\hat{b}}_{k}^{†}-\alpha_{k}^{*}\left(t\right){\hat{b}}_{k}\right]$ , and $\alpha_{k}\left(t\right)=2\frac{g_{k}}{\omega_{k}}\left(1-e^{i\omega_{k}t}\right)$ [46]. The associated master equation is given by

(15) $${\dot{\rho }}_{s}\left(t\right)=\frac{-i\tilde{\epsilon }\left(t\right)}{2}\left[\sigma_{z},\rho_{s}\left(t\right)\right]+\frac{\tilde{D}\left(t\right)}{2}\left[\sigma_{z}\rho_{s}\left(t\right)\sigma_{z}-\rho_{s}\left(t\right)\right],$$

where $\tilde{D}\left(t\right)=-\frac{d\ln(\left|\Gamma (t)\right|)}{dt}$ and $\tilde{\epsilon }\left(t\right)=-$ Im $[\frac{d\Gamma (t)/dt}{\Gamma (t)}].$

In the following, we consider that the bath starts from a squeezed thermal state [49,50,51,52]

(16) $$\rho_{B}\left(0\right)=\hat{\varsigma }\rho_{th}{\hat{\varsigma }}^{†},$$

where $\rho_{th}=e^{-\beta H_{B}}/Z_{\beta }$ is the thermal state with $Z_{\beta }$ the partition function; $\hat{\varsigma }$ $=\sum_{k}{\hat{s}}_{k}$ , where ${\hat{s}}_{k}=e^{\left[\left(s_{k}^{*}e^{-i\phi_{k}}{\hat{b}}_{k}^{2}-s_{k}e^{i\phi_{k}}{\left({\hat{b}}_{k}^{†}\right)}^{2}\right)/2\right]}$ is the squeezing operator for the boson bath mode ${\hat{b}}_{k}$ with $s_{k}$ and $\phi_{k}$ being the bath squeezing strength and phase, respectively. In this situation, the function $\Gamma \left(t\right)$ could be evaluated under the summation of the modes of the squeezed thermal bath as [46,47,48,49,50]

(17) $$\Gamma \left(t\right)=e^{\left[-\sum_{k}\frac{4{\left|g_{k}\right|}^{2}}{\omega_{k}^{2}}\left(1-\cos\omega_{k}t\right)\gamma_{k}\left(t\right)\coth\left(\frac{\omega_{k}}{2T}\right)\right]}$$

with $\gamma_{k}\left(t\right)=\cosh2s_{k}-\sinh2s_{k}\cos\left(\omega_{k}t-\Delta \phi_{k}\right)$ and $\Delta \phi_{k}$ is the phase difference between the squeezing phase $\phi_{k}$ relative to the phase of the coupling strength $g_{k}$ .

Substituting the coupling spectral density $J\left(\omega \right)=2\pi \sum_{k}{\left|g_{k}\right|}^{2}\delta \left(\omega -\omega_{k}\right)$ into Equation (17), we can transform the above summation in $\Gamma \left(t\right)$ into an integral for continuous bath modes as

(18) $$\begin{array}{ccc} \Gamma \left(t\right) & = & \exp\{-\int_{0}^{\infty }\frac{d\omega }{\pi \omega^{2}}2J\left(\omega \right)\left(1-\cos\omega t\right)\coth\left(\omega /2T\right)\\ & & \times \left[\cosh\left(2s\right)-\sinh\left(2s\right)\cos\left(\omega t-\Delta \phi \right)\right]\}. \end{array}$$

In the present work, we adopt the Ohmic coupling spectral density $J\left(\omega \right)=\eta \omega e^{-\omega /\varpi }$ with ϖ cutoff frequency, and η is the coupling strength [53]. Note that such engineering of the spectrum’s Ohmicity seems possible when simulating the dephasing model using a trapped ultracold atom, as demonstrated in reference [54,55]. In the high-temperature regime, the approximation coth(ω/2T)≈2T/ω has been taken, and in the case of ϖ>>T , the dephasing process is Markovian, and after straightforward algebra, one finds

(19) $$\Gamma \left(t\right)=e^{\left\{-\frac{2\eta Tt}{\pi }\left[\pi \cosh(2s)-\ln4\sinh(2s)\sin\Delta \phi \right]\right\}}.$$

Expression (Equation (19)) is the exact analytic for the time-dependent dephasing rate $\Gamma \left(t\right)$ in the present model.

**Figure 4:**
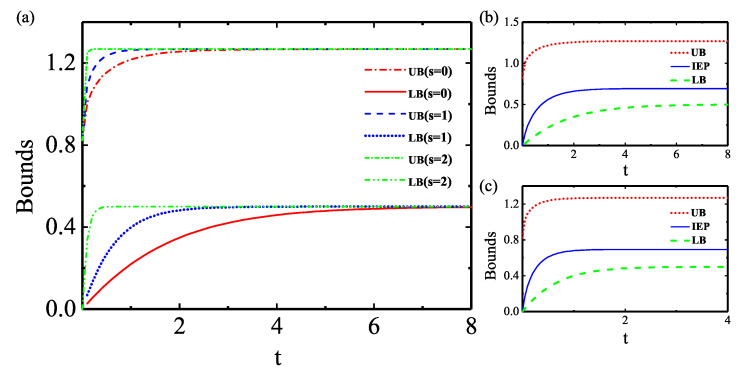
(**a**) Time dependence of LB and UB of IEP under different degrees of squeezing *s* in the dephasing model; time dependence of IEP (blue solid line) and its LB (red dotted line) and UB (green dashed line) in the case of (**b**) s=0 and (**c**) s=1 ; the initial state of the system is $\left(|e\rangle +|g\rangle \right)/\sqrt{2}$ , and the parameter Δϕ=π/4 .

Special attention was paid to the time dependence of the IEP as well as its geometrical bounds, as shown in Figure 4, where one can find that all the quantities exhibit asymptotic behaviors approaching their stationary values. They correspond to relaxations of the system through the dephasing channel due to the system–bath coupling. Regarding the time dependence of the bounds, we encounter another common feature that the equilibrium times of bounds decrease with the growing degree of squeezing. For instance, the equilibrium times for UB (LB) are 3 (7.3), 2.5 (3.3), and 0.4 (0.6) when s=0 , 1, and 2, respectively. However, unlike the dissipation model, both UB and LB increase monotonically as the squeezing character of the bath grows, and only the LB becomes tighter (compared with the thermal bath) during the whole dynamic process in the dephasing model. As a contrast, the tightness of LB only appears in the early stage of evolution for the dissipation model. By comparing the time evolutions of IEP with the two blue solid-lines in panel (b,c) of Figure 4, we find that the IEP is well located inside the region between the LB and the UB and reaches its stationary value faster with the increase of the squeezing parameter *s*, and the values of the IEP are reduced due to the existence of the squeezing effect.

**Figure 5:**
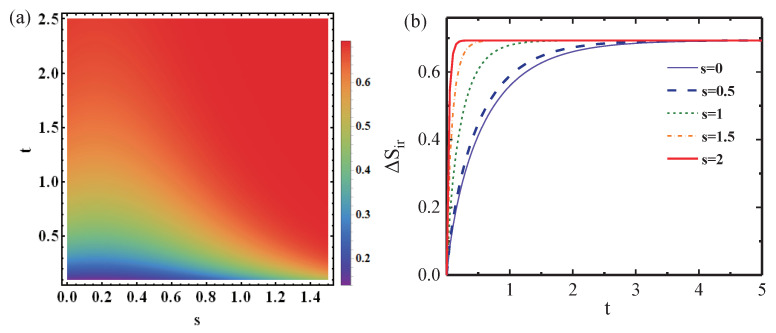
(**a**) The values of the IEP $▵S_{ir}$ in the parameter plane of {s,t} in the dephasing model; (**b**) time dependence of IEP $▵S_{ir}$ under the different degrees of squeezing *s*; here, the parameter Δϕ=π/4 .

In Figure 5, we provide numerical estimates of the IEP in the parameter space of {s,t} . Figure 5 tells us that one can precisely control the thermodynamic irreversibility through adjusting the parameters of the bath. As shown in Figure 5b, under the dephasing model, the IEP reaches equilibrium faster as the squeezed parameter increases, and the value of the IEP in longtime limit $△S_{irr}\left(\infty \right)$ will eventually converge together, irrespective of the values of parameter *s*. It means that in the dephasing model, the squeezing effect could not make too much impact on the thermodynamic irreversibility in the longtime limit, although the existence of squeezing drives the system into equilibrium faster. Physically, on a fundamental level, quantum coherence and the related dephasing process could also alter the possible state transitions in thermodynamic processes [56] and may even modify the fluctuation–dissipation relation [57,58] and quantum nonequilibrium work relation [59]. Additionally, when a system relaxes to equilibrium through contact with a thermal bath, quantum coherences are known to contribute an additional term to the IEP [60,61,62]. Different from the dissipation case where the system can exchange energy with its bath, in the dephasing model, this open system can never exchange energy with its bath. However, the information and correlation exchange between the system and the bath are dominant during the dynamics, and this exchange also influence the IEP. As a result, any alteration in the von Neumann entropy (basis of IEP and its bound) resulting from the relaxation process (dissipation or dephasing) has contributions not only from the change in population but also from decoherence. In this regard, it was pointed out that the entropy production can be split in two contributions, an incoherent one (stemming from populations) and a coherent one (stemming from quantum coherences) [63,64,65].

## 4. Discussions and Conclusions

The ideal physical system to verify our prediction is a linear optics system or quantum photonic simulator, as demonstrated in Ref. [11]. From an experimental point of view, one of the key elements in the present work is the squeezing thermal bath. Here, we could use the experimental approach of using coherent states to seed the parametric process that generates the bright squeezed states [66] or use the approach of applying the squeezing unitary on the input seed coherent states [67]. As an alternative, the universal and reversible low-loss broadband squeezer [68] has demonstrated the efficient and deterministic squeezing of a single photon [69]. Optical squeezing has been promoted from an offline experimental resource to a controllable online operation which is needed for our purposes. This work demonstrated that squeezing low-photon-number states has become an achievable feat, and it has lots of potential applications in quantum information processing and quantum thermodynamics. Therefore, the abovementioned technologies are highly suitable for the simulation of a squeezing thermal bath. On the other hand, the dissipation model can be realized by an amplitude damping channel through the qubits injected in the photonic setup which performs the logical operations [11], and the dephasing model can be realized using the approach in Ref. [70].

The study of IEP is of importance due to its intimate relation with the arrow of time in classical and quantum systems [71,72], the SLT [73,74,75], thermodynamic operations and thermal machines [76,77,78,79], and quantum and classical speed limits [8,80,81]. Therefore, tightening the bounds of IEP not only deepens our understanding of how much entropy production changes during the thermodynamic process but also provides insights into how to improve the performance of quantum thermodynamic devices. Additionally, the memory effect of non-Markovian dynamics in open quantum systems is often believed to be useful for quantum information processing. The reason is that a non-Markovian bath could be taken as a memory resource which could return the information of the open systems [82]. Therefore, the influence from the non-Markovian bath on the irreversibility of an open system is also interesting, which is the topic of our future work.

Interaction with a squeezed thermal bath is not the only generalized process that goes beyond the typical settings in classical thermodynamics. Our findings demonstrate how to utilize the squeezing effect of a bath as a resource to control the irreversibility, where the use of a nonthermal bath offers more degrees of control and manipulation, such as the amount of squeezing. Note that quantum bath engineering techniques have become powerful tools that enable the realization of arbitrary thermal and nonthermal baths. For instance, experimental realizations of squeezed thermal states range from superconducting circuit QED [83,84,85] to optomechanical mechanical oscillators [86,87]. The key parameters considered in our numerical simulation, such as the inverse temperature β and the degree of squeezing *s*, could be experimentally controlled using the current technologies demonstrated in the abovementioned experiments. Additionally, there have been many experiments focused on the assessment of nonequilibrium thermodynamic irreversibility using the technology of quantum trajectories of stochastic dynamics in nuclear magnetic resonance setups [3], superconducting qubit [88], and mechanical resonator [89], respectively. Our results reveal more detailed properties of thermodynamic irreversibility that are stronger than the conventional SLT for a given restricted class of irreversible processes. Along with other studies addressing squeezing effects in quantum thermodynamics, we hope that our analyses help to unveil the role of squeezing effects in quantum thermodynamic devices.

In summary, we studied the influence of squeezed characteristics of a bath on the IEP of open quantum systems. The results show that the equilibrium rates of IEP and its bounds become faster, and the values of IEP are reduced through harvesting the benefits of squeezing effects in the case of both a dissipation model and a dephasing model. In the dissipation model, the summarized overall trends of geometric bounds (LB and UB) found in reference [11] also hold for a squeezed thermal bath, while only the UB becomes tighter in the longtime limit, and the LB exhibits subtle tightness in the early stage of evolution compared to the thermal bath. Unlike the dissipation model, both UB and LB increase monotonically as the squeezing character of the bath grows, and only the LB becomes tighter (compared to the thermal bath) during the whole dynamic process in the dephasing model.

Moreover, the above-summarized trends for the bounds are independent of system size and hold for systems having more degrees of freedom. Our results do not contradict the SLT, which is modified by the inclusion of squeezing as an available resource in the bath. It is worth noting that a general evolution and the associated geometrical bounds of irreversibility of two-level system in thermal bath were theoretically analyzed and experimentally demonstrated in [11]. Here, we further highlight the role of adjustable parameters in bath, such as temperature and squeezing degree, on the reduction of thermodynamic irreversibility. It is expected that the present work helps in developing a better understanding of the irreversibility under ambient conditions.
