# Correction: Zou et al. Geometrical Bounds on Irreversibility in Squeezed Thermal Bath. *Entropy* 2023, *25*, 128

**DOI:** 10.3390/e27111097

**Published:** 2025-10-24

**Authors:** Chen-Juan Zou, Yue Li, Jia-Kun Xu, Jia-Bin You, Ching Eng Png, Wan-Li Yang

**Affiliations:** 1 Research Center of Nonlinear Science, School of Mathematical and Physical Science, Wuhan Textile University, Wuhan 430200, China; cjzou@hunnu.edu.cn; 2 State Key Laboratory of Magnetic Resonance and Atomic and Molecular Physics, Innovation Academy for Precision Measurement Science and Technology, Chinese Academy of Sciences, Wuhan 430071, China; liyue6683@gmail.com (Y.L.); ywl@wipm.ac.cn (W.-L.Y.); 3 School of Physical Sciences, University of Chinese Academy of Sciences, Beijing 100049, China; 4 Institute of High Performance Computing, Agency for Science, Technology, and Research (A*STAR), 1 Fusionopolis Way, #16-16 Connexis, Singapore 138632, Singapore; pngce@ihpc.a-star.edu.sg

In the published article [1], the author Yue Li needs to add a second affiliation. The updated second affiliation is as follows: School of Physical Sciences, University of Chinese Academy of Sciences, Beijing 100049, China. The authors state that the scientific conclusions are unaffected. This correction was approved by the Academic Editor. The original publication has also been updated.
